# Obstructive Small Bowel Metastasis from Uterine Leiomyosarcoma: A Case Report

**DOI:** 10.1155/2014/603097

**Published:** 2014-03-04

**Authors:** Mutahir A. Tunio, Mushabbab AlAsiri, Rasha M. Saleh, Shomaila Amir Akbar, Nagoud M. Ali, Mohamed Abdalazez Senosy Hassan

**Affiliations:** ^1^Radiation Oncology, King Fahad Medical City, Riyadh, Saudi Arabia; ^2^Radiation Oncology, Comprehensive Cancer Center, King Fahad Medical City, Riyadh 59046, Saudi Arabia; ^3^Medical Oncology, Comprehensive Cancer Center, King Fahad Medical City, Riyadh 59046, Saudi Arabia; ^4^Radiation Oncology, King Fahad Medical City, Riyadh 59046, Saudi Arabia; ^5^Anatomic Pathology, King Fahad Medical City, Riyadh 11525, Saudi Arabia; ^6^Clinical Oncology, Minia Oncology Center, Minia, Egypt

## Abstract

*Background.* Uterine leiomyosarcoma is a rare and aggressive gynecologic malignancy with an overall poor prognosis. Lungs, bones, and brain are common sites of metastases of uterine leiomyosarcoma. Metastases of uterine leiomyosarcoma to the small bowel are extremely rare, and only four case reports have been published to date. *Case presentation.* A 55-year-old Saudi woman diagnosed with a case of uterine leiomyosarcoma treated with total abdominal hysterectomy (TAH) and bilateral salpingooophorectomy (BSO) presented in emergency room after sixteen months with acute abdomen. Subsequent work-up showed a jejunal mass for which resection and end-to-end anastomosis were performed. Biopsy confirmed the diagnosis of small bowel metastasis from uterine leiomyosarcoma. Further staging work-up showed wide spread metastasis in lungs and brain. After palliative cranial irradiation, systemic chemotherapy based on single agent doxorubicin was started. *Conclusion.* Metastatic leiomyosarcoma of small bowel from uterine leiomyosarcoma is a rare entity and is sign of advanced disease. It should be differentiated from primary leiomyosarcoma of small bowel as both are treated with different systemic chemotherapeutic agents.

## 1. Introduction

Uterine leiomyosarcoma accounts for 3% of all uterine malignancies and it metastasize through hematogenous dissemination to the lungs, liver, bones, and the brain [1]. Small bowel is an extremely rare site of metastasis from uterine leiomyosarcoma [2, 3]. Leiomyosarcoma of the small bowel can either be primary or secondary. Primary leiomyosarcomas arise from the muscularis propria layer of the intestinal wall and show immunopositivity DC117, CD34, and CK AE1/AE3. In contrary, secondary leiomyosarcomas arise from the mucosal layer of the intestinal wall and are negative for DC117, CD34, and CK AE1/AE3 [4]. Uterine leiomyosarcoma can invade small bowel by direct extension, hematogenous route, implantation, or via lymphatic route [5]. Acute abdomen (small bowel obstruction or perforation) secondary to metastatic uterine leiomyosarcoma to small bowel is not well known and has been documented by only four case reports till date [6]. Small bowel metastasis in uterine leiomyosarcoma is usually a sign of extensive disease and poor prognosis.

Herein we report a case of a 55-year-old Saudi women who presented in emergency room with acute abdomen secondary to small bowel metastasis from uterine leiomyosarcoma.

## 2. Case Presentation

A 55-year-old Saudi woman was admitted to the Emergency Department with a chief complaint of episodes of paroxysmal abdominal pain. The pain started two weeks previously that was aggravated for the last two days with nausea, vomiting, and constipation. Past medical and surgical history revealed that she was diabetic since last 12 years and was on oral metformin 500 mg three times a day and she underwent total abdominal hysterectomy (TAH) and bilateral salpingooophorectomy (BSO) sixteen months back for uterine leiomyosarcoma outside of our hospital without any adjuvant treatment.

**Figure 1:**
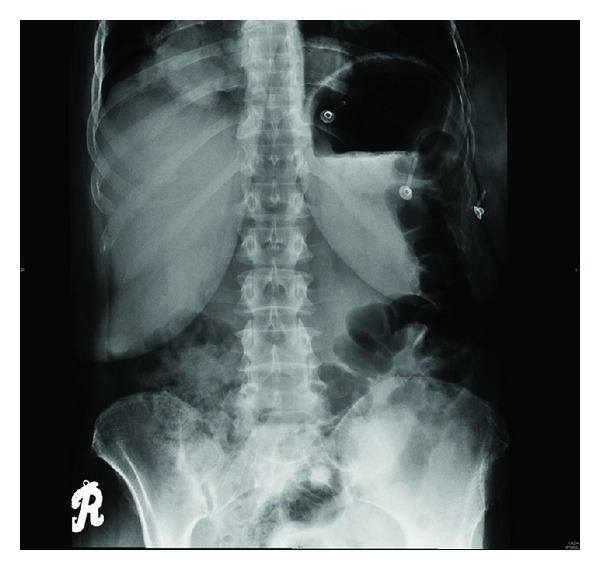
Plain abdominal radiograph showing dilatation of small bowel without any gas-fluid levels.

**Figure 2:**
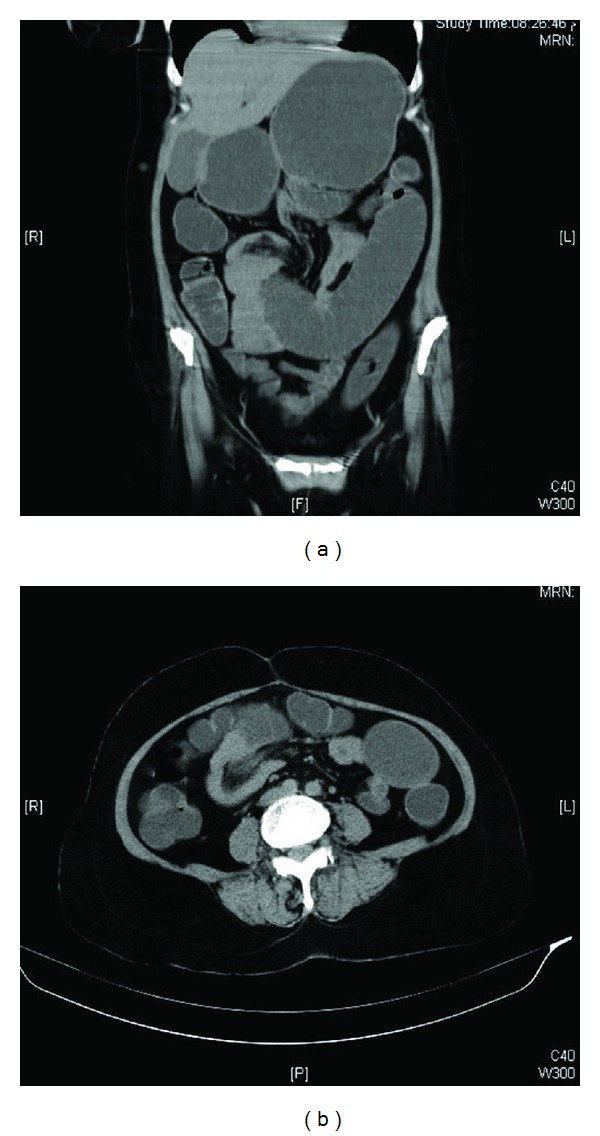
Computed tomography (CT) abdomen without contrast showing (a) coronal view: distended stomach, proximal small bowel loops extending down to midabdominal level where there was abrupt change in caliber of the bowel, and collapse of distal small bowel loops and (b) axial view: a soft tissue density lesion containing vessels.

**Figure 3:**
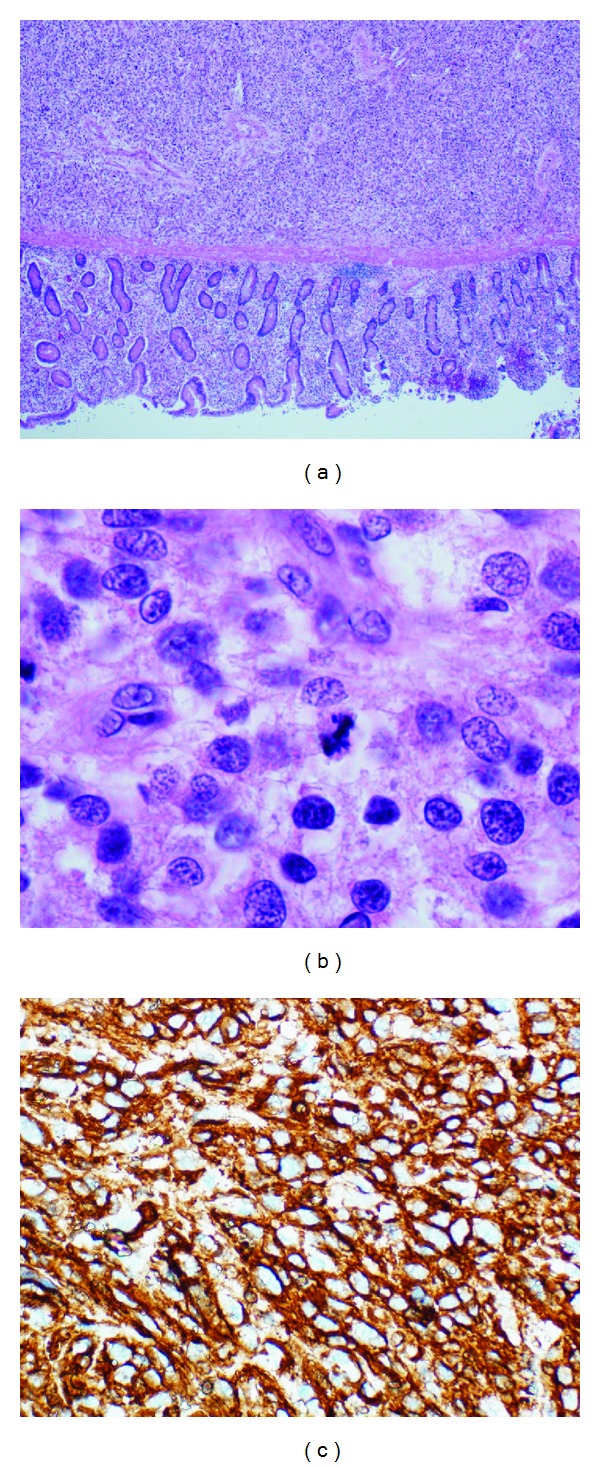
(a) Hematoxylin and Eosin staining (×40) (b) (×100) of the tumor at the submucosa and muscular layer of the small bowel and showing (c) immunopositivity for SMA.

**Figure 4:**
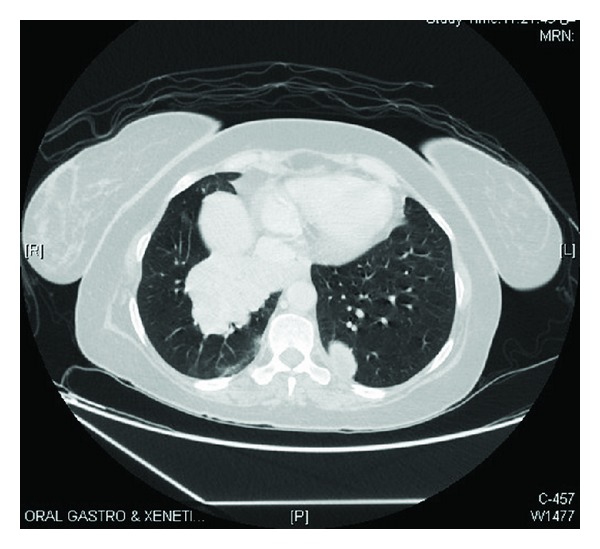
Computed tomography of chest showing largest pulmonary metastatic nodule of size 10 × 6 cm in right middle and lower lobes invading adjacent mediastinum, mediastinal pleura, pericardium, and right pulmonary vein and bronchus.

During physical examination, moderate tenderness over the right epigastric and hypochondrial areas and active bowel sounds were observed. Plain abdominal X-ray showed dilatation of small bowel without any gas-fluid levels; Figure 1. Baseline hematology showed leukocytosis (12.4 × 10^9^/L), high absolute neutrophil count (10.4 × 10^9^/L), and normal hemoglobin and platelets counts. Serum chemistry showed lactate dehydrogenase (LDH) was 237 U/L ↑ (normal: 135–214), sodium 132 mmoL/L ↓ (normal: 135–145), serum creatinine was 226 *μ*moL/L ↑ (normal: 44–80), urea 15.1 mmoL/L ↑ (normal: 2.5–6.4), anion gap was 31.2 mmoL/L (normal: 12–20), and lactate was 2.3 mmoL/L (normal: 1.1–2.2). Computed tomography (CT) abdomen without contrast showed distended stomach, proximal small bowel loops extending down to midabdominal level where there was abrupt change in caliber of the bowel, and collapse of distal small bowel loops. At this transition point appeared a soft tissue density containing vessels, which raised the possibility of intussusception with a lesion; Figure 2. Patient underwent exploratory laparotomy on the same day. During exploration, a jejunal mass of size 6 cm was found which was resected and anastomosis was performed. Histopathology of jejunal mass was leiomyosarcoma arising from mucosa. Further immunohistochemistry (IHC) favored jejunal mass as metastatic leiomyosarcoma rather than primary leiomyosarcoma (positivity for SMA and negativity for S100, DC117, CD34, and CK AE1/AE3); Figure 3. Further histopathological review of primary surgery specimen showed 7 cm uterine leiomyosarcoma involving out half of myometrium without any lymphovascular invasion (LVSI) which confirmed the diagnosis; Figure 4.

**Figure 5:**
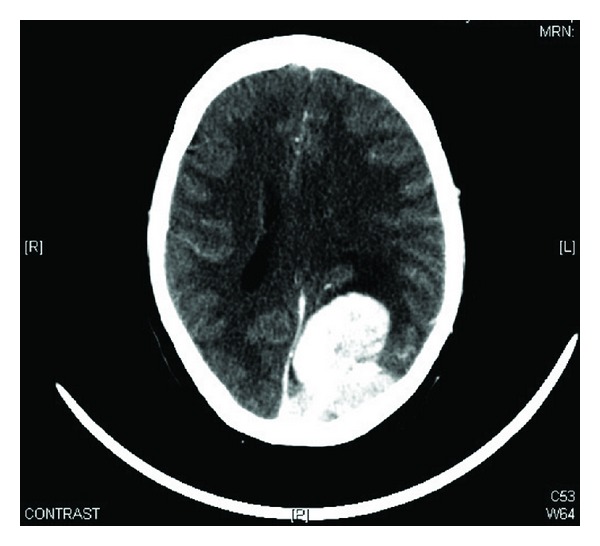
Computed tomography of brain showing multiple intra- and extracranial enhancing lesions at left occipital, left parietal subdura (5.6 × 3.5 cm) with midline shift and vasogenic edema, and right cerebellum.

After her successful postoperative recovery, staging workup was done. Bone scintigraphy was found normal. CT chest showed multiple pulmonary metastatic nodules; largest one was of size 10 × 6 cm in right middle and lower lobes invading adjacent mediastinum, mediastinal pleura, pericardium, and right pulmonary vein and bronchus; Figure 5. CT brain revealed multiple intra- and extracranial enhancing lesions at left occipital, left parietal subdura (5.6 × 3.5 cm) with midline shift and vasogenic edema, and right cerebellum. Also there was superior sagittal sinus tumoral thrombosis.

After discussing her case in multidisciplinary board meeting, she was given palliative cranial radiotherapy 20 Gy in 5 fractions over a week and then she was started on systemic chemotherapy based on single agent doxorubicin (60 mg/m^2^).

At time of publication patient was found alive and was receiving chemotherapy.

## 3. Discussion

Small bowel metastases are uncommon and account for 10% of all small bowel tumors [7]. The most common sites of primary tumor metastasizing to small bowel are adenocarcinoma of uterus, cervix, colon, and lung; ductal carcinoma of breast; and melanoma [8]. Metastasis to small bowel by uterine leiomyosarcoma is an extremely rare manifestation and carries an unfavorable prognosis. Only four cases have been reported so far in the world literature; Table 1.

The mechanism of metastasis to the heart is not well known. Possible routes are direct extension, hematogenous route, implantation, or via lymphatic route. In our patient small bowel was affected through hematogenous pathway. Metastatic leiomyosarcoma shall be differentiated from rare primary intestinal leiomyosarcoma with help of IHC; both are treated with different chemotherapeutic agents [4]. Primary leiomyosarcomas are uncommon tumors that arise from the muscularis propria layer of the intestinal wall in contrary to metastatic leiomyosarcomas which are mostly of mucosal origin [9].

Metastatic small bowel leiomyosarcoma secondary to uterine leiomyosarcoma is considered a sign of advanced disease with high tumor burden as seen in our patient. Such patients have shown response to doxorubicin alone, gemcitabine alone, or gemcitabine plus docetaxel with median survival rates of 14.7 to 24 months [10].

In conclusion, small bowel metastasis following treatment for uterine leiomyosarcoma is rare. IHC helps to differentiate it from primary leiomyosarcoma of small bowel. Metastatic leiomyosarcoma is sign of advanced disease with grave prognosis and such patients may benefit from single agent doxorubicin-based systemic chemotherapy.

## Figures and Tables

**Table 1:** Small bowel metastasis secondary to uterine leiomyosarcoma reported from 1993 to 2012.

Author	Age years	From time of initial treatment	Location	Other sites of metastasis	Treatment	Survival
Gerst et al. [2]	56	8 years after TAH + BSO	Ileoileal junction	Lungs Liver	Surgical resection chemotherapy	24 months

Ben-Ishay et al. [3]	60	4 years after TAH + BSO	Jejunum	Not mentioned	Surgical resection	Not mentioned

Sidani et al. [4]	48	5 years after TAH + BSO	Ileum	Lungs	Surgical resection chemotherapy	Not mentioned

Saylam et al. [6]	59	16 months after TAH + BSO	Ileoileal junction	Bones	Surgical resection	Alive at 10 months

Present case	57	16 months after TAH + BSO	Jejunum	Lungs Brain	Surgical resection, radiotherapy, and chemotherapy	Alive at 3 months

TAH + BSO: total abdominal hysterectomy and bilateral salpingooophorectomy.
